# Beyond the baby schema: Objects being touched are perceived to be cute

**DOI:** 10.1371/journal.pone.0340903

**Published:** 2026-02-19

**Authors:** Akane Ohashi, Hiroshi Nittono

**Affiliations:** Graduate School of Human Sciences, The University of Osaka, Suita, Osaka, Japan; Wadia Institute of Himalayan Geology, INDIA

## Abstract

Research on cuteness has traditionally focused on the baby schema, which is used to describe the physical characteristics of an individual. Recent research, however, has shown that the characteristics of interactions between individuals, such as physical contact and social relationships, can also be perceived as cute (or *kawaii* in Japanese) and evoke a special feeling associated with its perception. In this study, we aimed to examine the influence of seeing an object being touched by another person on impressions of cuteness or kawaii by manipulating the level of the baby schema features. Online surveys were conducted in Japan (*n* = 198) and the United States (*n* = 199). Participants were presented with four photographs depicting a person holding or not holding an object with high or low baby schema features and were asked to rate their feelings of cuteness or kawaii toward the object. In addition, following the procedures of previous research, they were asked to report their impression of the person in the photograph and their guess about how the person felt about the object. The results showed that the objects with higher baby schema features and the objects being touched were rated as cuter or more kawaii. These effects did not interact. Similar effects were observed for the cuteness ratings of the person and guesses about the person’s feelings. The present study suggests that the perceived cuteness of an object is influenced not only by its baby schema characteristics but also by factors such as physical contact or the relationship between the entities.

## Introduction

The concept of cuteness has attracted many researchers across various disciplines. To date, research on cuteness has been conducted in the fields of psychology [1–3], neuroscience and physiology [4–7], arts and humanities [8–10], marketing and social media [11–13], and engineering [14,15].

Cuteness is often understood as “infant physical attractiveness” [16], and many studies have examined responses to things related to infants. Lorenz proposed the concept of the *Kindchenschema*, or baby schema [17]. Like other species showing instinctive responses to certain physical attributes, humans react to a set of physical characteristics by perceiving them as cute and exhibiting affiliative and nurturing behaviors. The baby schema includes a large head in relation to body size, big eyes below the center of the skull, a large forehead, and short limbs. Subsequent studies have validated the concept by measuring facial features that are perceived as cute or by experimentally manipulating these physical attributes. For instance, infant faces that are perceived as cute exhibit high baby schema features [18–20], and faces with enhanced baby schema features increase perceived cuteness [21–24]. Although most research on cuteness has centered around the baby schema, the generalizability of this idea is still controversial [25].

One of the important features of cuteness is that it evokes a special kind of affective state related to caregiving, sometimes referred to as *tenderness* [26] or *nurturant love* [27]. Since this was first described by Lorenz [17], several researchers have argued for the use of different terms such as “cuteness response” [3], “kawaii” (Japanese word corresponding to cute or depicting the feeling associated with the perception of cuteness [2]), “aww” [28], and “kama muta” (positive emotion, meaning “moved by love” in Sanskrit [29]). Nittono proposed that the feeling of kawaii is elicited by the cognitive appraisal of the relationship between the observer and the object rather than by the perception of the baby schema or other cuteness cues of the object itself [2]. This theoretical framework posits that the perception of cuteness requires not only the physical characteristics of an object but also its surrounding context.

Following the tradition of the baby schema, most research on cuteness has focused on the physical appearance of an individual. However, recent research has revealed that the perception of cuteness is also influenced by factors other than the baby schema, such as the relationship between individuals. Steinnes et al. reported that the perceived cuteness of animals increased when people saw a communal sharing relationship between them [29]—a relationship characterized by closeness and equivalence, and known to elicit kama muta [30,31]. Videos depicting a high level of communal sharing (e.g., humans and/or small animals playing with and feeding each other) were rated as cuter than those depicting a low level of communal sharing. Similarly, Shiomi et al. demonstrated that objects (e.g., children, small humanoid robots, or cherries) facing and touching each other were perceived to be cuter than those that were not [32]. Using video clips in which a human or humanoid robot model introduced a stuffed doll to the participants, Okada et al. examined whether the presenter’s touch on the doll affected the viewers’ impressions [33]. They reported that (1) the doll was rated as cuter when it was touched by the model than when it was not, (2) the participants guessed that the model found the doll cuter when the model was touching the doll than when they were not, and (3) the perceived cuteness of the model was not affected by the touching action. These findings suggest that the perception of cuteness, or the feeling of *kawaii*, for multiple entities is influenced by the perception of their mutual relationship, such as physical contact.

### Present research

In the present study, we systematically manipulated the level of the baby schema features of an object and its relation to another person and examined how these factors affected perceived cuteness. Previous research has demonstrated that social relationships between individuals influence the perception of cuteness [29,32,33]. However, these studies only considered objects with high baby schema features. We attempted to replicate and expand the findings that an object was perceived to be cuter when touched by a person by manipulating the object’s physical appearance. Additionally, to control the stimulus attributes more strictly, we used still pictures rather than video clips. Following the protocol of Okada et al. [33], we asked participants about how cute they found the object was, their impressions of the person who touched (or did not touch) the object, and their guesses about how that person felt about it.

In addition, three further issues were investigated. First, we also considered dimensions other than cuteness, infantility and beauty, to test whether the effect is specific to the perception of cuteness. Although the ratings of cuteness and infantility have a moderate positive correlation, it has been reported that their distributions differ [2,34], and not all cute objects have infantile features (e.g., confectionery, accessories, and smiles [35]). Similarly, although cuteness and beauty are both types of attractiveness [16,36], their core meanings are different [37]. Adult female faces perceived to be cute and those perceived to be beautiful are associated with distinct facial features [38].

Second, the survey was conducted in both Japan and the United States to examine the generalizability of the findings. Because the response to cute things is considered universal across cultures [29,37,39], similar results were expected in both countries, regardless of whether the word “kawaii” or “cute” was used.

Third, the effect of personality differences in empathy was exploratorily investigated. Several studies have indicated that a high empathic concern trait relates high sensitivity to cuteness [29,40–42]. Therefore, the level of the empathic concern trait could correlate with the level of perceived cuteness reported in this study.

This experiment was preregistered before sampling (https://osf.io/xa4gc for the study in Japan, https://osf.io/zcu9k for the study in the United States). The preregistered hypotheses were as follows:

**H1**. The feeling of kawaii (or cuteness) will be greater for an object with high baby schema features than for an object with low baby schema features.

**H2**. The observer’s feeling of kawaii toward an object will be greater when the model is touching the object than when they are not.

**H3**. The effect of seeing an object being touched on the feeling of kawaii will be greater for the object with high baby schema features than for the object with low baby schema features.

**H4.** The feeling of kawaii toward the model will be unaffected by their touching action.

**H5**. The participants will guess that the model feels that the object is more kawaii when the model is touching it than when they are not.

**H6**. The rating of kawaii will show a different pattern than the ratings of infantility and beauty.

## Methods

### Participants

#### Japan.

Given a two-tailed paired *t*-test, power analyses using G*power [43] resulted in *N* = 54 (*d*_z_ = 0.5, α = .05, 1 − β = .95) or *N* = 90 (*d*_z_ = 0.3, α = .05, 1 − β = .80). For an exploratory analysis of individual differences in empathic traits, the required sample size was doubled (*N* = 200). Participants were recruited from Lancers (Lancers, Inc., Tokyo, Japan), an online crowdsourcing service. According to a preregistered procedure, to remove invalid answers, participants were excluded if they met any of the following criteria: those who answered all questions with the same rating value, those who answered their age and sex differently at the beginning and end of the survey, and those who had missing responses. As a result, two people whose ages were inconsistent were excluded from the analysis. Data from the remaining 198 participants (116 male and 82 female, *M =* 44.92 years old, age range 21–72) were used to test the hypotheses. Participants had self-reported normal or corrected-to-normal visual acuity and were required to be native Japanese speakers. The protocol was approved by the Behavioral Research Ethics Committee of the University of Osaka School of Human Sciences, Japan (HB023-094R), and electronic informed consent was obtained from all participants. The research began and concluded on November 17, 2023. Participants received 120 Japanese yen as monetary compensation via Lancers, Inc.

#### United States.

The same study with the same stimulus images, questions, sample size, and exclusion criteria was conducted in English for native English speakers with United States citizenship. 202 participants were recruited from Prolific (Prolific Academic Ltd., London, England). Three people whose age or gender were inconsistent were excluded from the analysis. Data from the remaining 199 participants (112 male, 84 female, and 3 other, *M* = 41.65 years old, age range 19–75) were used to test the hypotheses. Participants received $1.40 USD as monetary compensation via Prolific. The protocol was approved by the Behavioral Research Ethics Committee of the University of Osaka School of Human Sciences, Japan (HB023–144), and electronic informed consent was obtained from all participants. The research began and concluded on December 21, 2023.

### Stimuli

**Fig 1:**
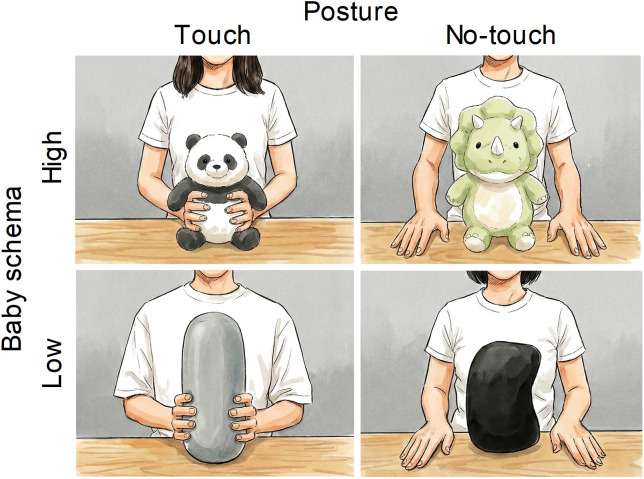
Illustrations of stimuli based on photographic data used for the four conditions. From the top left, a high baby schema object is touched by a model, a high baby schema object is not touched by a model, a low baby schema object is touched by a model, and a low baby schema object is not touched by a model. Four different human models appeared in the experiment. Four different human models appeared in the experiment. The four illustrations presented here were created by Gemini 3 Pro Image (Google LLC) using photographs taken by one of the authors [Akane Ohashi] as visual reference prompts. The shapes of the objects were altered by the authors from the originals. According to Google’s policy on the Use of Generated Content (https://ai.google.dev/gemini-api/terms#use-generated), this figure is available under the Creative Commons Attribution 4.0 International (CC BY 4.0) license.

Fig 1 shows examples of the stimuli used in the present study. Four types of photographs were prepared: the object’s baby schema (high or low) × the model’s posture (touch or no touch). Two stuffed animals (a panda and a triceratops) were used as high baby schema objects, and two cylindrical pillows (black and gray) were used as low baby schema objects. All objects were purchased from Daiso Industries Co., Ltd. (Higashi-Hiroshima, Japan). Two men and two women served as models. They either held the object in their hands or placed their hands on the table. Their faces did not appear in the picture. A total of 32 photographs (4 objects × 2 postures × 4 models) were taken (see https://osf.io/9yb64 for all photographs). All photos were 438 × 328 pixels in size to ensure smooth loading and display in the online surveys. Thirty-two sets of photos were created by combining four images, with the constraint that each object or model appeared only once and that the images of each factor contained both male and female models.

### Procedure

The survey was conducted online using the Qualtrics platform (Qualtrics, LLC., Provo, Utah, United States). The participants were required to complete the experiment on a personal computer (PC) to ensure that the rating screen was displayed correctly, and they were instructed to continue without taking a break in the middle of the experiment. They first provided informed consent and information about their age and sex. The rating task was then explained, followed by a practice session.

**Fig 2:**
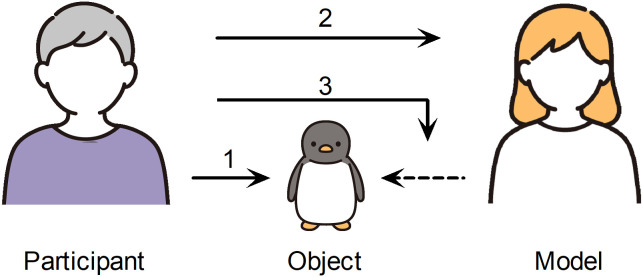
Schematic illustration of the rating targets. The participants were asked to rate (1) their impression of the object, (2) their impression of the model, and (3) their guess about the model’s impression of the object. They rated each of the targets from the perspectives of cuteness/*kawaii,* infantility/*osanai,* and beauty/*utsukushii*.

In the rating task, the participants were asked to rate their impressions of each photo on a 7-point scale (*kawaii*, *osanai*, and *utsukushii* in Japan; cute, infantile, and beautiful in the United States; 1 = *not at all,* 7 = *extremely*) with a mouse click. They were told that the term “impression” meant a personal “sense” or “feeling” and were asked to answer honestly, without thinking too deeply, as there were no correct answers. Three ratings were given in the following order: (1) the object, (2) the model, and (3) a guess about the model’s impression of the object (see Fig 2). Each participant was randomly assigned one of 32 stimuli sets, and they responded 9 times per photo, for a total of 36 responses. The orders of the four conditions and rating dimensions were randomized across participants.

There was only one practice trial; no pictures were presented, and participants were asked to respond with the median of the three rating dimensions. After completing the rating task, the participants completed an empathy scale. In Japan, we used the multi-dimensional empathy scale [44], which measured empathic traits and consisted of four subscales: empathic concern, personal distress, fantasy, and cognitive empathy. They responded to 30 items on a 5-point scale (1 = *does not describe me well,* 5 = *describes me very well*). In the United States, participants’ empathic traits were measured with the Interpersonal Reactivity Index [45], which consists of 28 items on a 5-point scale (0 = *does not describe me well,* 4 = *describes me very well*).

### Statistical analysis

First, according to the preregistered protocol, a two-way repeated measures analysis of variance (ANOVA) with the factors of object’s baby schema (high or low) and model’s posture (touch or no touch) was conducted on the ratings separately for each country. Three rating targets and three rating dimensions were analyzed separately. The significance level was set at 0.05. To compare the effect sizes of the object’s baby schema and that of the model’s posture, each *d* was calculated by dividing the difference between the means of the two conditions by the standard deviation pooled across the two conditions [46].

For an explanatory analysis, Pearson’s correlation coefficients (*r*) between the ratings of cuteness/kawaii and empathic traits were calculated. Empathic trait ratings were calculated for each subscale. The calculation of three cuteness/kawaii scores was performed for each rating target: overall (i.e., the average across the four conditions), the difference between the high and low conditions of the object’s baby schema, and the difference between the touch and no-touch conditions of the model’s posture. To compensate for multiple comparisons, the false discovery rate of a total of 36 tests (i.e., 4 empathic traits subscale, 3 cuteness/kawaii ratings, and 3 rating targets) was controlled for at 0.05 [47]. All analyses were conducted using JASP version 0.95.2 [48] for ANOVAs and R statistical software version 4.4.3 [49] for adjusting the *p*-value of the correlation analyses.

## Results

### Japan

**Fig 3:**
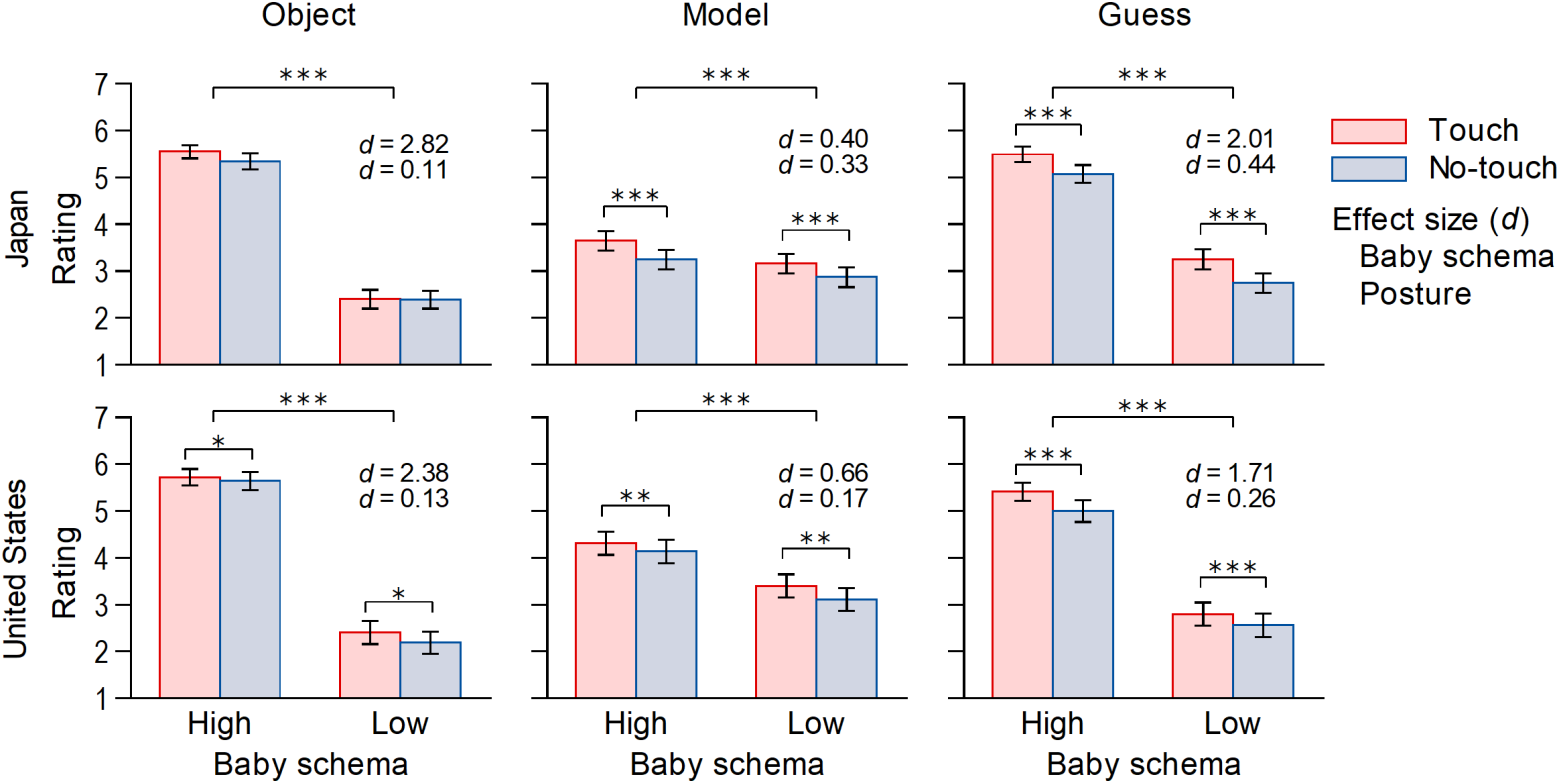
Mean cuteness/kawaii ratings of the three rating targets. Error bars indicate 95% confidence intervals. *p < .05, **p < .01, ***p < .001.

Fig 3 shows the mean cuteness/kawaii ratings of the three rating targets for each condition. The distribution of the ratings is shown in S1 Fig in the supplementary material. In accordance with the pre-registration, Object’s Baby Schema × Model’s Posture ANOVAs were conducted on the ratings. Table 1 shows the results of the ANOVAs for cuteness/kawaii ratings. There was a significant main effect of the object’s baby schema on the object’s kawaii ratings. The main effect of the model’s posture and the interaction effect were not significant, although the mean value was higher when the model was touching the object than when they were not. The main effects of the object’s baby schema and the model’s posture were significant for both the kawaii ratings of the model and the participant’s guess about the model’s impression of the object. No interaction was found. The results of the osanai and utsukushii ratings were similar to those of the kawaii ratings (S2 and S3 Figs and S1 Table in the supplementary material).

**Table 1:** Summary of the Object’s Baby Schema × Model’s Posture ANOVAs on cuteness/kawaii ratings.

		Japan				United States			
		*F*	*p*		η_p_^2^	*F*	*p*		η_p_^2^
Object	Baby schema	1158.50	<.001	***	.86	614.37	<.001	***	.76
	Posture	2.42	.121		.01	5.42	.021	*	.03
	Baby schema × Posture	2.45	.119		.01	1.42	.235		.01
Model	Baby schema	25.31	<.001	***	.11	80.67	<.001	***	.29
	Posture	20.08	<.001	***	.09	8.95	.003	**	.04
	Baby schema × Posture	0.20	.652		.00	0.23	.633		.00
Guess	Baby schema	522.33	<.001	***	.73	349.15	<.001	***	.64
	Posture	34.76	<.001	***	.15	16.15	<.001	***	.08
	Baby schema × Posture	0.31	.576		.00	1.38	.242		.01

The numerator and denominator degrees of freedom for all *F*-tests were 1 and 197 in Japan and 1 and 198 in the United States, respectively. *p < .05, **p < .01, ***p < .001.

As part of the exploratory analysis, Pearson’s correlation coefficients were calculated for the correlations between the ratings of kawaii and empathic traits. No significant correlations were observed (see S2 Table in the supplementary material).

### United States

The distribution of the ratings is shown in S4 Fig in the supplementary material. There were significant main effects of the object’s baby schema and the model’s posture on the object’s cuteness ratings. The interaction effect was not significant. In addition, the results of both the cuteness ratings of the model and the participant’s guess about the model’s impression of the object were the same as those of the object ratings. The results of the infantility and beauty ratings were similar to those of the cuteness ratings. Pearson’s correlation coefficients were calculated for the correlations between the cuteness ratings and empathic traits for the exploratory analysis. No significant correlations were observed between them.

### Omnibus analyses of Japan and the United States

Although not preregistered, the data from the two countries were combined and analyzed together, including the country as a factor. Table 2 shows the results of the Country × Object’s Baby Schema × Model’s Posture ANOVAs on the ratings for cuteness/kawaii. There were significant main effects of the object’s baby schema and the model’s posture on the object’s cuteness/kawaii ratings. The main effect of the country was not significant, and no interaction effect was found. All the main effects on the model’s cuteness/kawaii ratings were significant. The interaction effects of Country × Model’s Posture and Country × Object’s Baby Schema × Model’s Posture were not significant. The main effects of the object’s baby schema and the model’s posture on the participants’ guess ratings were significant, while the main effect of the country was not. The interaction effects of Country × Model’s Posture and Country × Object’s Baby Schema × Model’s Posture were not significant. The results of the infantility/osanai and beauty/utsukushii ratings were similar to those of the cuteness/kawaii ratings (see S3 Table in the supplementary material).

**Table 2:** Summary of the Country × Object’s Baby Schema × Model’s Posture ANOVAs on the cuteness/kawaii ratings in the omnibus analysis.

	Object				Model				Guess			
	*F*	*p*		η_p_^2^	*F*	*p*		η_p_^2^	*F*	*p*		η_p_^2^
Country	0.51	.474		.00	21.81	<.001	***	.05	3.82	.051		.01
Baby schema	1548.25	<.001	***	.80	103.19	<.001	***	.21	817.57	<.001	***	.67
Posture	7.37	.007	**	.02	27.81	<.001	***	.07	48.85	<.001	***	.11
Country × Baby schema	4.30	.039	*	.01	15.32	<.001	***	.04	2.04	.154		.01
Country × Posture	0.16	.692		.00	1.01	.316		.00	1.49	.224		.00
Baby schema × Posture	0.11	.745		.00	0.00	.987		.00	0.14	.714		.00
Country × Baby schema× Posture	3.83	.051		.01	0.43	.512		.00	1.44	.230		.00

The numerator and denominator degrees of freedom for all *F-*tests were 1 and 395, respectively. *p < .05, **p < .01, ***p < .001.

## Discussion

The purpose of this study was to examine whether the perceived cuteness of an object was affected by its being touched by another person. The baby schema features of the object were also manipulated. The results showed that the objects with higher baby schema features and the objects being touched were rated as cuter, without interaction between these factors. Additionally, the participants rated the model as cuter and guessed that the model perceived the object as cuter when the model touched the object. Infantility/osanai and beauty/utsukushii ratings showed similar effects. These findings were consistent in both Japan and the United States.

Our findings support both H1 (The feeling of kawaii will be greater for an object with high baby schema features than for an object with low baby schema features) and H2 (The observer’s feeling of kawaii toward an object will be greater when the model is touching the object than when they are not). These effects align with previous findings on the perception of cuteness, both for the baby schema [21–24] and physical contact between objects and other entities [29,32,33]. These results suggest that perceived cuteness is affected not only by its attributes but also by its surrounding context. The similar results in Japan and the United States suggest that the effect of physical contact on the feeling of kawaii was not culture specific. However, it should be noted that the effect size for an object being touched was much smaller (*d*s = 0.11 in Japan and 0.13 in the United States) than for an object having high baby schema features (*d*s = 2.82 in Japan and 2.38 in the United States). A similar small effect size was also reported in Okada et al.’s study [33] (*d* = 0.15 in Japanese participants). Therefore, an individual’s appearance is considered a dominant factor in cuteness perception.

Given that the level of the baby schema features largely determine perceived cuteness, the effect of physical touch was expected to be greater for the objects with high baby schema features. Although this was what we originally predicted (H3: The effect of seeing an object being touched on the feeling of kawaii will be greater for the object with high baby schema features than for the object with low baby schema features), no statistically significant interaction was found. One possible reason for this is that the effect of the model’s touching posture was too small to produce an interaction effect. Another possibility is that these factors, the baby schema and physical contact, may affect cuteness perception additively. Touch is considered to play a role in emotional communication [50,51] and can convey altruistic feelings (i.e., love, gratitude, and sympathy [52,53]). Physical contact as a representation of social affiliation could be another cue for perceived cuteness, separate from the baby schema, and could have effects that are independent of an individual’s appearance. Previous studies have shown that factors such as children’s personality [54,55], the content of their statements [56], and the perceivers’ cultural background [57] affect cuteness perception. This study provides an additional example of cuteness cues beyond an individual’s appearance.

When the model touched the object, the participants rated the model as cuter and guessed that the model perceived the object as cuter, which did not support H4 (The feeling of kawaii toward the model will be unaffected by their touching action), but supported H5 (The participants will guess that the model feels that the object is more kawaii when the model is touching it than when they are not). These results indicate that observing a model touching an object influences not only how cute the object is perceived to be but also how cute the model is perceived to be and their guess about the model’s impression. Although the present study did not support H4, which was based on Okada et al.’s findings [33], it is consistent with their original hypothesis (i.e., The presenter’s touching behavior enhances the feeling of kawaii toward the presenter). This inconsistency may be due to the availability of the model’s facial or vocal cues, which have been demonstrated to be important for a person’s impression [58,59]. In the study by Okada et al. [33], video stimuli were used, and participants saw the model’s face and heard their voice. These explicit cues may have determined the perceived cuteness of the model and obscured the small effect of the model’s posture (touch or no touch). These cues were not available in the present study, which may have led to the small effect being statistically significant.

There were no notable disparities in the patterns among the similar adjectives. This result did not support H6 (The rating of kawaii will show a different pattern than the ratings of infantility and beauty). In previous studies demonstrating that infantility and beauty are distinct from cuteness, participants were asked to rate them in different blocks [37,38,60] or they were rated by another person [34,61]. By contrast, the participants in this study evaluated the three adjectives on a single rating page, which may have produced superficial correlations between the ratings. Because the mean rating values differed across the three adjectives (see S2 and S3 Figs), participants should have rated each adjective differently. However, the distinction in meaning may not be as clearcut as initially presumed.

The similar results in Japan and the United States suggest that the effect of physical contact on the feeling of kawaii was not culture specific. The general response to cute things is considered universal across cultures [37,39], and mutual affective behavior can cross-culturally amplify feelings of “kama muta,” a construct linked to perceived cuteness [29]. Our results add the evidence that physical contact between two entities also influences the perception of cuteness cross-culturally.

In this study, no statistically significant correlations were observed between cuteness ratings and empathic traits. This result is inconsistent with previous research [29,40–42]. The difference may be because the participants in this study evaluated the perceived cuteness of adults, pillows, and stuffed toys. Kanai and Nittono reported that, although a higher level of empathy was associated with an increased perception of cuteness in babies and inanimate objects (e.g., clothing and accessories), no predictive relationships were observed in the perceptions of cuteness in animated characters (e.g., illustrations and stuffed toys depicting living creatures) [40]. In a similar vein, Takamatsu reported that empathic concern was correlated with responses to the cuteness of human and animal babies but not correlated with responses to animated objects such as character goods [42]. The perceived cuteness measured in this study may not be associated with empathy.

### Limitations and future directions

Although this study was able to replicate and expand the findings of Okada et al. [33], there are several limitations. First, although this study indicates a small but significant effect of physical contact on perceived cuteness, it did not provide a definitive answer about how an individual’s baby schema features impact this effect. Further research could explore this interaction by employing more impactful forms of contact, such as mutual contact [29,32]. Second, the ecological validity of the experimental environment was low in this study (e.g., the absence of voice and facial expression) due to its aim to strictly control stimulus attributes. Given the association between smiling and perceiving something as cute [35,60,62,63], it is conceivable that people’s facial expressions and touching posture may interactively affect participants’ perceived cuteness and their guess about the person’s feeling about the object. Third, the choice of the two types of objects with high and low baby schema features (i.e., stuffed animals and pillows) was arbitrary. Different results could be obtained by using other types of objects, such as dolls that resemble either babies or adults. However, since the current stimulus type already had a large effect on perceived cuteness, it is unlikely that the overall results will change.

The present study focused exclusively on the subjective ratings of cuteness or kawaii. If kawaii is an emotion, it can be measured from three perspectives: subjective, physiological, and behavioral [2]. The baby schema induces prolonged gazing and smiling [35,62,63] and increased careful and prosocial behavior [64–66]. It would be worthwhile to ascertain in future studies whether the observation of physical contact also induces these behavioral and physiological responses.

## Conclusion

The present study, conducted in Japan and the United States, demonstrated that an object with high baby schema features or that is touched by another individual is perceived as cuter and showed that these effects are additive. Our results provide another example of how the perception of an object’s cuteness is influenced by both its physical baby schema features and the surrounding context. A more comprehensive approach to cuteness and kawaii is warranted beyond the traditional view of the baby schema.

## Supporting information

S1 Fig Distribution of the ratings for each rating dimension and target in Japan. (DOCX)

S2 Fig Mean infantility/osanai ratings of the three rating targets. (DOCX)

S3 Fig Mean beauty/utsukushii ratings of the three rating targets. (DOCX)

S4 Fig Distribution of the ratings for each rating dimension and target in the United States. (DOCX)

S1 Table Summary of the Object’s Baby Schema × Model’s Posture ANOVAs on infantility/osanai and beauty/utsukushii ratings. (DOCX)

S2 Table Pearson’s correlation coefficients between individual empathic traits and the cuteness/kawaii ratings. (DOCX)

S3 Table Summary of the Country × Object’s Baby Schema × Model’s Posture ANOVAs on infantility/osanai and beauty/utsukushii ratings. (DOCX)
