# Supplementary figures and images for: Beyond the baby schema: Objects being touched are perceived to be cute
Source: PLoS One. 2026 Feb 19;21(2):e0340903. doi: 10.1371/journal.pone.0340903 (PMC12919793; doi:10.1371/journal.pone.0340903)

**S1 Fig. Distribution of the Ratings for Each Rating Dimension and Target in Japan.**

**
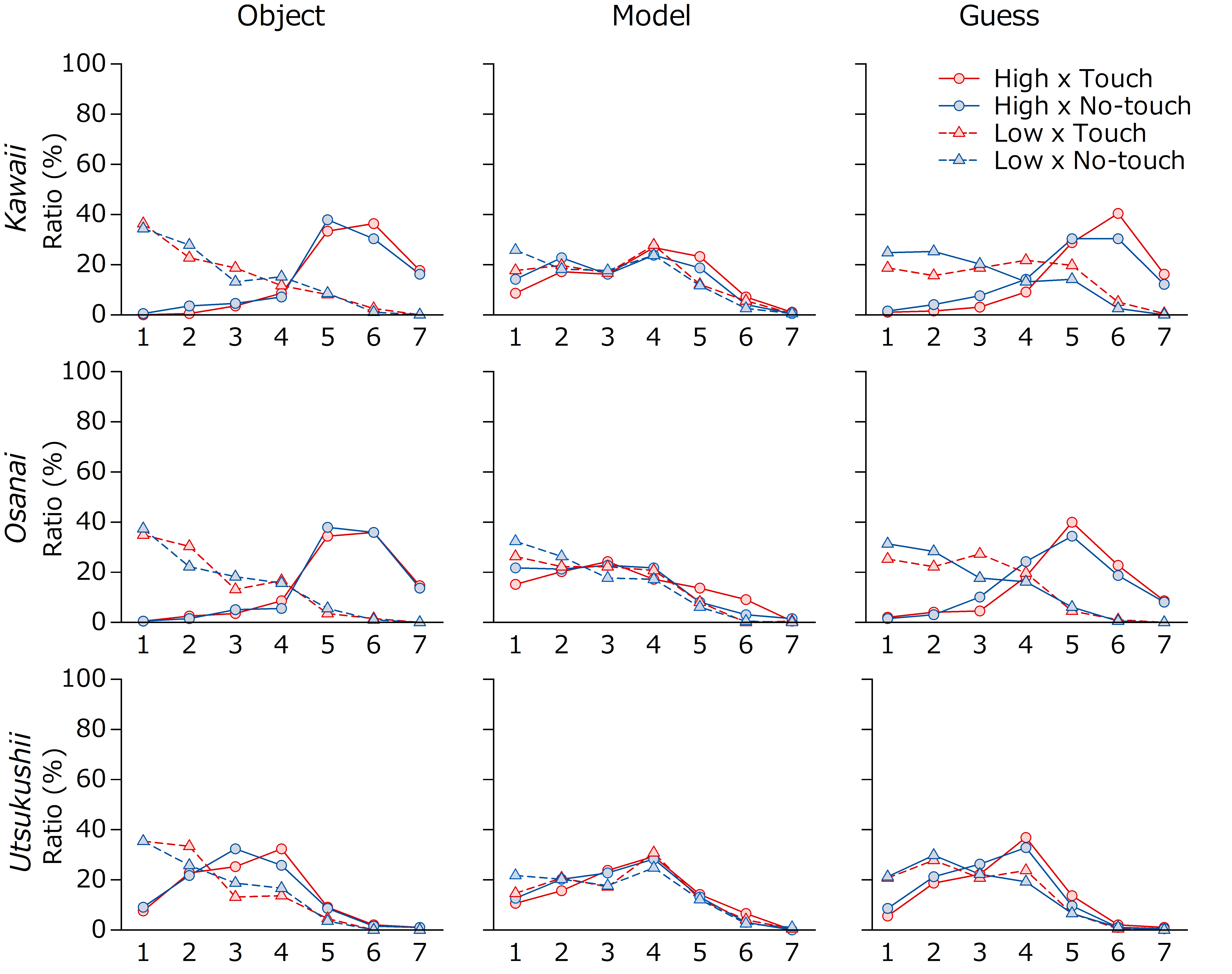
**

Supplement: S1 Fig — (DOCX) [file pone.0340903.s001.docx]

**S4 Fig. Distribution of the Ratings for Each Rating Dimension and Target in the United States.**


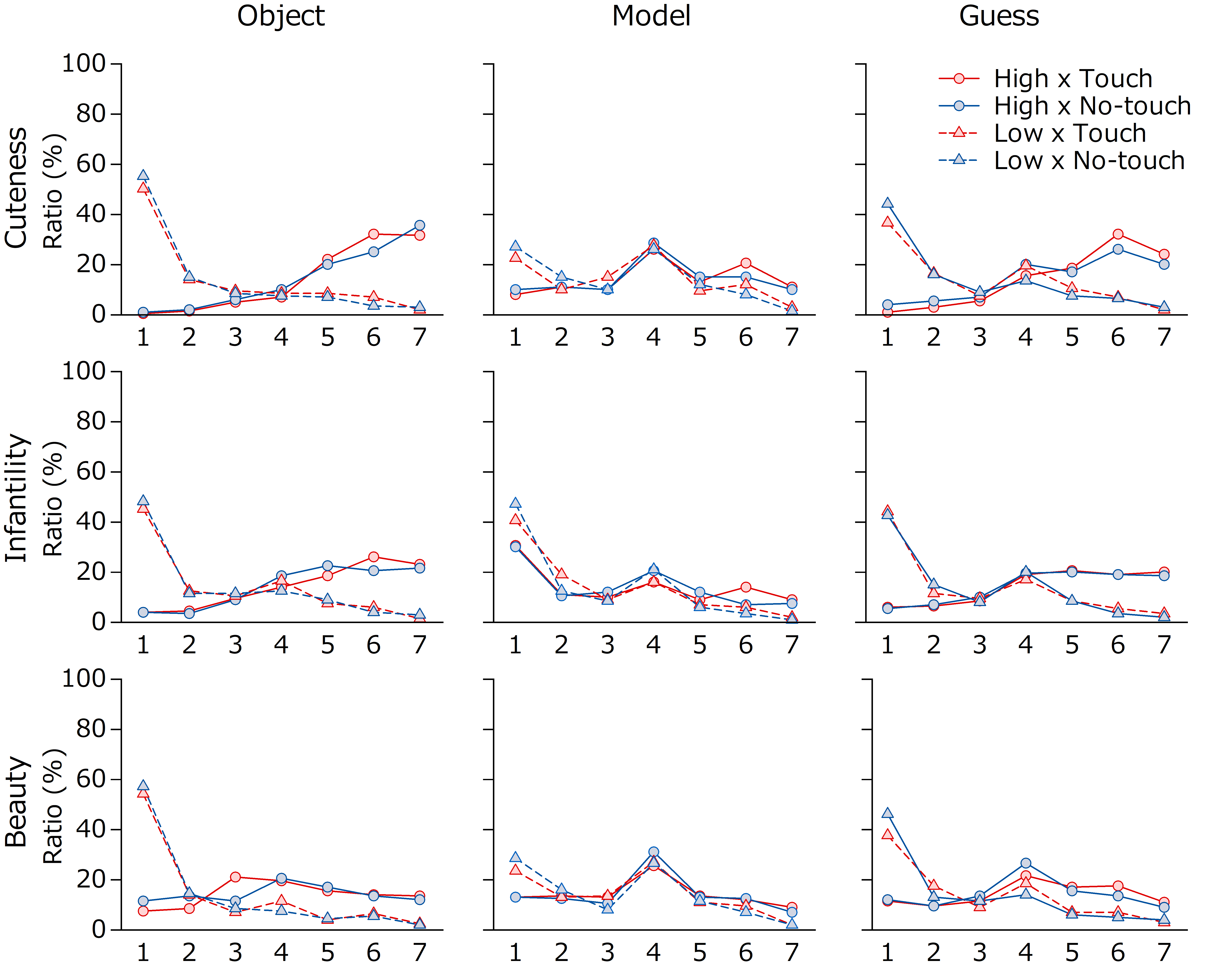

Supplement: S4 Fig — (DOCX) [file pone.0340903.s004.docx]
