# Supplementary material for: Beyond the baby schema: Objects being touched are perceived to be cute
Source: PLoS One. 2026 Feb 19;21(2):e0340903. doi: 10.1371/journal.pone.0340903 (PMC12919793; doi:10.1371/journal.pone.0340903)
Supplement: S2 Fig — (DOCX) [file pone.0340903.s002.docx]

**S2 Fig. Mean Infantility/Osanai Ratings of the Three Rating Targets.**


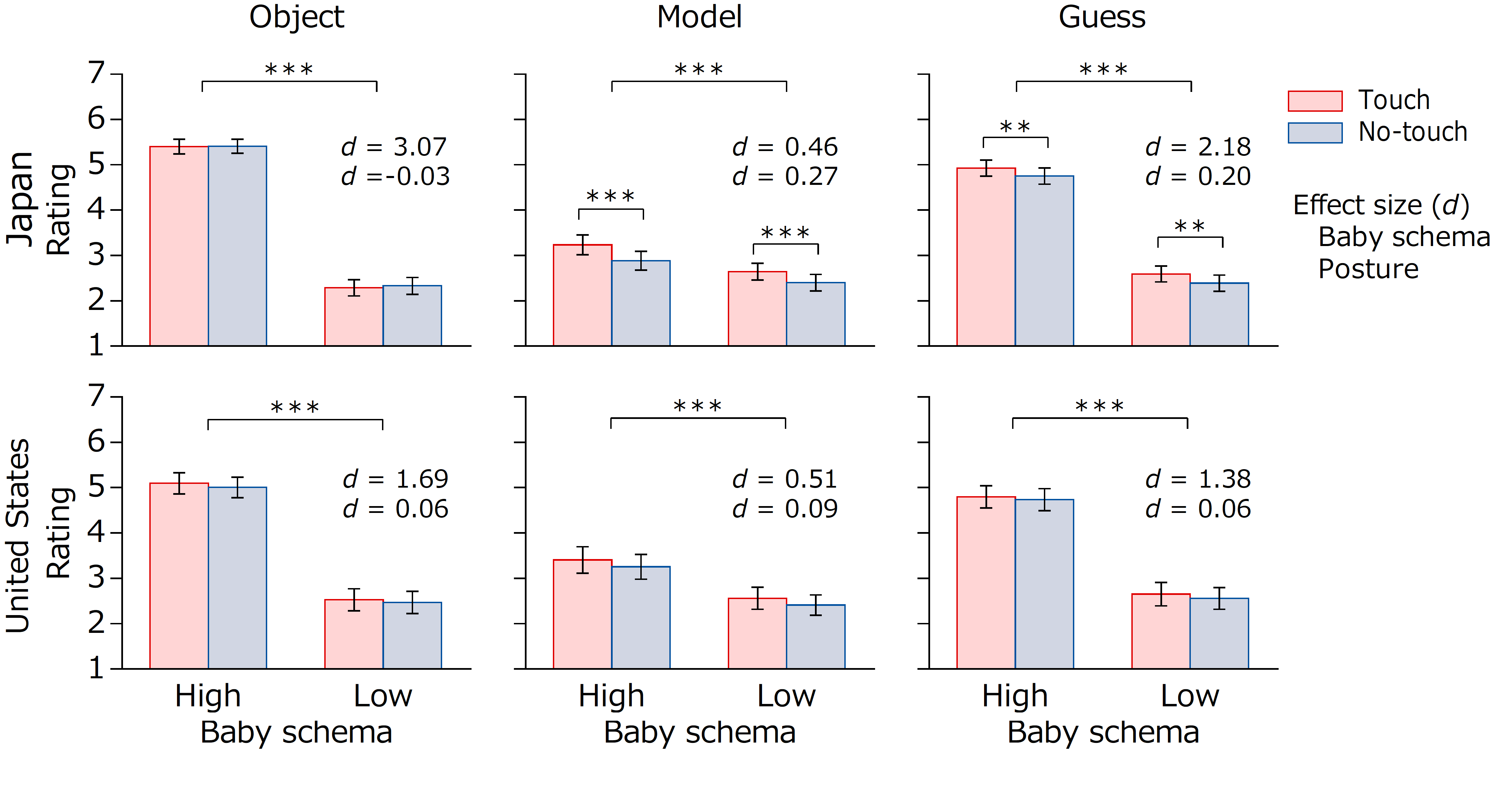


Error bars indicate 95% confidence intervals. **p* < .05, ***p* < .01, ****p* < .001.
