# Supplementary material for: Beyond the baby schema: Objects being touched are perceived to be cute
Source: PLoS One. 2026 Feb 19;21(2):e0340903. doi: 10.1371/journal.pone.0340903 (PMC12919793; doi:10.1371/journal.pone.0340903)
Supplement: S1 Table — (DOCX) [file pone.0340903.s005.docx]

**S1 Table. Summary of the Object’s Baby Schema × Model’s Posture ANOVAs on Infantility/Osanai and Beauty/Utsukushii Ratings.**

|  |  | Japan | | | | United States | | | | | |  |
| --- | --- | --- | --- | --- | --- | --- | --- | --- | --- | --- | --- | --- |
| Infantility/Osanai | | *F* | *p* |  | η_p_^2^ | *F* | *p* | |  | | η_p_^2^ |  |
| Object | Baby schema | 1064.38 | <.001 | *** | .84 | 326.39 | <.001 | *** | | .62 | |  |
|  | Posture | 0.17 | .679 |  | .00 | 0.97 | .326 |  | | .01 | |  |
|  | Baby schema × Posture | 0.06 | .801 |  | .00 | 0.04 | .846 |  | | .00 | |  |
| Model | Baby schema | 38.72 | <.001 | *** | .16 | 58.79 | <.001 | *** | | .23 | |  |
|  | Posture | 17.07 | <.001 | *** | .08 | 3.13 | .078 |  | | .02 | |  |
|  | Baby schema × Posture | 0.36 | .548 |  | .00 | <.001 | 1.000 |  | | .00 | |  |
| Guess | Baby schema | 536.55 | <.001 | *** | .73 | 221.38 | <.001 | *** | | .53 | |  |
|  | Posture | 7.48 | .007 | ** | .04 | 1.06 | .305 |  | | .01 | |  |
|  | Baby schema × Posture | 0.06 | .814 |  | .00 | 0.05 | .823 |  | | .00 | |  |
|  | | Japan | | | | United States | | | | | |  |
| Beauty/Utsukushii | | *F* | *p* |  | η_p_^2^ | *F* | *p* | |  | | η_p_^2^ | |
| Object | Baby schema | 137.01 | <.001 | *** | .41 | 270.88 | <.001 | | *** | | .58 | |
|  | Posture | 0.05 | .829 |  | .00 | 7.70 | .006 | | ** | | .04 | |
|  | Baby schema × Posture | 2.66 | .105 |  | .01 | 0.03 | .864 | |  | | .00 | |
| Model | Baby schema | 9.61 | .002 | ** | .05 | 49.50 | <.001 | | *** | | .20 | |
|  | Posture | 10.21 | .002 | ** | .05 | 2.22 | .137 | |  | | .01 | |
|  | Baby schema × Posture | <.001 | 1.000 |  | .00 | 0.89 | .347 | |  | | .00 | |
| Guess | Baby schema | 72.06 | <.001 | *** | .27 | 188.16 | <.001 | | *** | | .49 | |
|  | Posture | 6.20 | .014 | * | .03 | 7.25 | .008 | | ** | | .04 | |
|  | Baby schema × Posture | 2.32 | .130 |  | .01 | 0.02 | .880 | |  | | .00 | |

The numerator and denominator degrees of freedom for all *F* tests were 1 and 197 in Japan and 1 and 198 in the United States, respectively. **p* < .05, ***p* < .01, ****p* < .001.
