# Supplementary material for: Beyond the baby schema: Objects being touched are perceived to be cute
Source: PLoS One. 2026 Feb 19;21(2):e0340903. doi: 10.1371/journal.pone.0340903 (PMC12919793; doi:10.1371/journal.pone.0340903)
Supplement: S2 Table — (DOCX) [file pone.0340903.s006.docx]

**S2 Table. Pearson’s Correlation Coefficients Between Individual Empathic Traits and the Cuteness/Kawaii Ratings.**

| Object | Japan | | | | United States | | | |
| --- | --- | --- | --- | --- | --- | --- | --- | --- |
|  | EC | FS | PD | PT | EC | FS | PD | PT |
| Overall | .220 | .114 | .071 | .077 | .044 | -.012 | .130 | .128 |
| Baby schema | .114 | .067 | -.036 | .042 | .108 | .237 | -.073 | -.088 |
| Posture | -.087 | -.122 | -.017 | -.216 | -.034 | -.059 | -.019 | .002 |
| Model | Japan | | | | United States | | | |
|  | EC | FS | PD | PT | EC | FS | PD | PT |
| Overall | .095 | .011 | .116 | .024 | .024 | .008 | .191 | .136 |
| Baby schema | .084 | -.007 | -.031 | .123 | .090 | .056 | -.045 | .013 |
| Posture | -.110 | -.169 | .029 | -.134 | -.081 | .019 | .001 | .001 |
| Guess | Japan | | | | United States | | | |
|  | EC | FS | PD | PT | EC | FS | PD | PT |
| Overall | .106 | .023 | .054 | .043 | -.036 | -.089 | .159 | .041 |
| Baby schema | .087 | <.001 | -.095 | .015 | .061 | .191 | -.069 | -.144 |
| Posture | .060 | -.103 | -.054 | .008 | .099 | .185 | .056 | .073 |

EC, PD, FS, and PT represent Empathic Concern, Personal Distress, Fantasy, and Perspective-taking, respectively. Overall refers to the mean rating value collapsed across the four conditions. Baby schema refers to the difference between the high and low conditions of the object’s baby schema. Posture refers to the difference between the touch and no-touch conditions of the model’s posture. The false discovery rate for a total of 36 tests (i.e., for each country, 4 empathic trait subscales, 3 cuteness/kawaii ratings, and 3 rating targets) was controlled at 0.05, using the method of Benjamini and Yekutieli [47]. No significant correlations were observed in either Japan or the United States.
