# Supplementary material for: Beyond the baby schema: Objects being touched are perceived to be cute
Source: PLoS One. 2026 Feb 19;21(2):e0340903. doi: 10.1371/journal.pone.0340903 (PMC12919793; doi:10.1371/journal.pone.0340903)
Supplement: S3 Table — (DOCX) [file pone.0340903.s007.docx]

**S7 Table. Summary of the Country × Object’s Baby Schema × Model’s Posture ANOVAs on Infantility/Osanai and Beauty/Utsukushii Ratings.**

|  | Object | | | | Model | | | | Guess | | | |
| --- | --- | --- | --- | --- | --- | --- | --- | --- | --- | --- | --- | --- |
| Infantility/Osanai | *F* | *p* |  | η_p_^2^ | *F* | *p* |  | η_p_^2^ | *F* | *p* |  | η_p_^2^ |
| Country | 0.70 | .402 |  | .00 | 0.84 | .361 |  | .00 | 0.03 | .860 |  | .00 |
| Baby schema | 1099.69 | <.001 | *** | .74 | 97.36 | <.001 | *** | .20 | 646.76 | <.001 | *** | .62 |
| Posture | 0.22 | .640 |  | .00 | 16.08 | <.001 | *** | .04 | 6.73 | .010 | ** | .02 |
| Country × Baby schema | 10.26 | .001 | ** | .03 | 4.78 | .029 | * | .01 | 1.09 | .297 |  | .00 |
| Country × Posture | 1.03 | .311 |  | .00 | 1.69 | .194 |  | .00 | 1.14 | .286 |  | .00 |
| Baby schema × Posture | 0.10 | .754 |  | .00 | 0.20 | .653 |  | .00 | 0.10 | .748 |  | .00 |
| Country × Baby schema × Posture | <.001 | .980 |  | .00 | 0.20 | .653 |  | .00 | <.001 | .981 |  | .00 |
|  | Product | | | | Model | | | | Guess | | | |
| Beauty/Utsukushii | *F* | *p* |  | η_p_^2^ | *F* | *p* |  | η_p_^2^ | *F* | *p* |  | η_p_^2^ |
| Country | 15.41 | <.001 | *** | .04 | 7.46 | .007 | ** | .02 | 9.44 | .002 | ** | .02 |
| Baby schema | 406.89 | <.001 | *** | .51 | 56.86 | <.001 | *** | .13 | 258.68 | <.001 | *** | .40 |
| Posture | 4.34 | .038 | * | .01 | 10.91 | .001 | ** | .03 | 13.42 | <.001 | *** | .03 |
| Country × Baby schema | 50.07 | <.001 | *** | .11 | 16.07 | <.001 | *** | .04 | 38.89 | <.001 | *** | .09 |
| Country × Posture | 3.13 | .077 |  | .01 | 1.38 | .240 |  | .00 | 0.18 | .670 |  | .00 |
| Baby schema × Posture | 1.66 | .198 |  | .00 | 0.39 | .534 |  | .00 | 0.91 | .341 |  | .00 |
| Country × Baby schema × Posture | 1.10 | .295 |  | .00 | 0.39 | .534 |  | .00 | 1.37 | .243 |  | .00 |

The numerator and denominator degrees of freedom for all *F*-tests were 1 and 395, respectively. **p* < .05, ***p* < .01, ****p* < .001.
